# Flow cytometry-based validation of soluble biomarker detection

**DOI:** 10.1016/j.plabm.2025.e00510

**Published:** 2025-11-07

**Authors:** Yiting Tang, Xiang Wu, Huating Zhang, Liu Dong, Ruoshui Cao, Jian Chen, Jiajun Zhu, Lianlong Hu, Qingyu Zhou, Jianming Zhou, Ke Qian, Yong Lin, Shuying Chen

**Affiliations:** a Department of Laboratory Medicine, Huashan Hospital, Fudan University, Shanghai, China; b Shanghai Medical College, Fudan University, Shanghai, China; c Department of Clinical Laboratory, Central Laboratory, Jing'an District Center Hospital of Shanghai, Fudan University, Shanghai, China; d Department of Laboratory Medicine, Shanghai Fifth People's Hospital, Fudan University, Shanghai, China

**Keywords:** Flow cytometry, sCD25, sCD40L, sCD130, sTREM-1, BD FACSLyric, Soluble markers, Immune response

## Abstract

**Objectives:**

Accurate and reliable measurement of soluble markers, such as sCD25, sCD40L, sCD130, and sTREM-1, is crucial for understanding their roles in immune responses and inflammatory conditions. This study presents the validation of a multiplex flow cytometry assay using the BD FACSLyric flow cytometer, designed to simultaneously quantify these four soluble markers in serum samples.

**Methods:**

The assay utilizes a bead-based immunoassay kit that is compatible with BD FACSLyric to enable precise detection of low concentrations of these markers. The methodological approach includes detailed sample preparation, bead conjugation, and assay optimization. Data acquisition was performed on the BD FACSLyric, with a minimum of 10 000 events recorded per sample to ensure robust data collection.

**Results:**

The validation results demonstrated that the assay achieved low limits of detection (LOD) for sCD25, sCD40L, sCD130, and sTREM-1. The LOD values were respectively 9.77 pg/ml, 43.95 pg/ml, 219.73 pg/ml, and 12.21 pg/ml, indicating the assay's high sensitivity. Reproducibility was confirmed through intra-assay and inter-assay variability assessments. Studies have shown that the levels of sCD25/sCD40L/sTREM-1 in the body are associated with lung cancer. Therefore, this study also used this detection method to test both lung cancer patients and healthy individuals, finding significant differences in the results, which can be used to assist in clinical diagnosis and treatment.

**Conclusions:**

This assay's ability to detect multiple analytes in a single run, combined with its high sensitivity and reproducibility, makes it a valuable asset for both research and clinical applications. Future work may expand this assay to include additional markers or adapt it for other sample types, further enhancing its utility in diverse biomedical contexts.

## 1 Introduction

Soluble markers such as sCD25, sCD40L, sCD130, and sTREM-1 play crucial roles in regulating immune responses [1,2]. sCD25, also known as soluble interleukin-2 receptor alpha (sIL-2Rα), is a marker of T cell activation [3]. The levels of sCD25 are significantly elevated in the clinical progression of various diseases, such as autoimmune diseases, infectious diseases, certain contagious diseases, and organ transplant rejection reactions, among others [[4], [5], [6]]. sCD40L is the soluble form of the CD40 ligand. CD40^−^CD40L is an important pair of co-stimulatory molecules in the specific immune response system within the body, involved in both humoral and cellular immune responses [[7], [8], [9]]. sCD40L is mainly distributed at the junction of atherosclerotic plaques and normal tissues [10,11], serving as a marker of immune system activation [11], and is associated with autoimmune and chronic inflammatory diseases [12]. sCD130 is a soluble protein produced by the cleavage of the IL-6 receptor, acting as a negative regulator [13]. It mediates IL-6/IL-6R signal transduction and is involved in immune response, hematopoiesis, inflammation, as well as the functions of the endocrine and nervous systems [[14], [15], [16]]. sTREM-1 is the extracellular domain fragment of soluble TREM-1, which plays a significant role in the inflammatory response and cascade amplification effect in infectious diseases. sTREM-1 is specifically expressed on the surface of neutrophils and monocytes [17]. Upon stimulation by various pathogens, such as bacteria and viruses, its expression level significantly increases and is released, making it one of the serum detection indicators for the presence of infection in the human body [18]. During the pathogenesis of inflammatory infectious diseases, the level of sTREM-1 in the blood or body fluids will significantly rise [19,20].

The pursuit of reliable soluble biomarkers for cancer diagnosis and prognosis is an active area of research, as evidenced by our group's previous work on serum exosomal microRNAs in hepatocellular carcinoma [21] and the functional role of miRNAs like miR-19a-3p [22] and miR-200a [23,24] in tumor progression. Accurate and reliable quantification of these soluble markers is essential for understanding their roles in disease mechanisms and for developing potential diagnostic and therapeutic strategies. Traditional methods for measuring soluble markers, such as enzyme-linked immunosorbent assays (ELISA), often involve individual assays for each marker, which can be time-consuming and require large sample volumes. In contrast, flow cytometry offers a powerful alternative by enabling the simultaneous detection and quantification of multiple analytes from a single sample, thereby improving efficiency and throughput.

In this study, we aimed to validate a multiplex flow cytometry assay using the BD FACSLyric flow cytometer for the simultaneous measurement of sCD25, sCD40L, sCD130, and sTREM-1. The BD FACSLyric system is well-suited for this application due to its high sensitivity, multi-color detection capabilities, and ease of use. We utilized a bead-based immunoassay kit compatible with the BD FACSLyric to facilitate the multiplex detection of these markers.

The validation process involved optimizing the assay conditions, including sample preparation, bead conjugation, and detection procedures. We assessed the assay's performance through a series of validation parameters, including sensitivity, specificity, and reproducibility. The goal is to establish a robust and reliable experimental method that can be used for research and clinical applications, providing auxiliary diagnostic information on the state of the organism's immune system.

By addressing these objectives, this study provides a valuable tool for comprehensive analysis of soluble biomarkers, aimed at providing a reliable and reproducible method for immune monitoring, applicable in both research and clinical settings to guide therapeutic decisions and improve patient outcomes.

## 2 Materials and methods

### 2.1 Research design and patient sample

This study aims to develop and validate a detection method based on flow cytometry, using the BD FACSLyric flow cytometer to measure the levels of sCD25, sCD40L, sCD130, and sTREM-1 in serum samples. Serum samples from 100 lung cancer patients and 100 healthy controls were collected. The inclusion criteria for patients included a diagnosis of lung cancer and availability of blood samples. All participants provided informed consent, and the study was approved by the ethics committee of Huashan Hospital, Fudan University, in accordance with, and the Declaration of Helsinki.

### 2.2 Reagents and instruments

Flow Cytometer: The BD FACSLyric flow cytometer was used for data acquisition, equipped with lasers and detectors capable of detecting multiple fluorescence channels simultaneously (Table SI1).

Detection Kit: Use the sCD25/sCD40L/sCD130/sTREM-1 combined detection kit (flow cytometry fluorescence luminescence method) compatible with BD FACSLyric. This kit is produced by Jiangxi Saiji Biotechnology Co., Ltd., and consists of capture microsphere mixture, detection antibody reagent, SA-PE, sample diluent, wash buffer (10 × ), calibrator, matrix B, and quality control materials.

Plasma Sample: Human plasma samples were collected from healthy donors and patients with various inflammatory conditions. Samples were stored at −20 °C until use.

### 2.3 Sample data collection

Preparation of washing buffer: Stabilize the 10 × washing buffer to room temperature until all salts are dissolved; Add 5 ml of 10 × washing buffer to 45 ml of purified water.

Sample Preparation: Dilute 25 μL of thawed serum sample with sample diluent at a 1:1 ratio. Prepare the standards and quality control samples according to the manufacturer's instructions. All samples are prepared in duplicate or triplicate to ensure accuracy.

Addition of capture microsphere mixture: Add 25 μL of capture microsphere mixture to the diluted serum samples, standards, and quality control samples respectively. Mix thoroughly and vortex for 1 min.

Detection antibody and fluorescent dye addition: Add 25 μL of detection antibody to all tubes, vortex and mix for 2–3 s. Then shake in the dark at room temperature for 2 h; subsequently add 25 μL of SA-PE to all tubes, vortex and mix for 2–3 s, then shake in the dark at room temperature for 0.5 h.

Data collection: Add 1 ml of wash buffer to each tube, and resuspend the microspheres by vortexing for 2–3 s. Centrifuge at 250g for 5 min, discard the supernatant, then add 150–300 μL of wash buffer to each tube, resuspend the microspheres by vortexing, and collect data using a BD FACSLyric flow cytometer. Record at least 10 000 events per sample to ensure statistical reliability.

### 2.4 Gating strategy

For the BD FACSLyric detection of soluble markers, we employed a detailed gating strategy to ensure accurate and reliable data analysis. Beads were first identified using FSC-A vs SSC-A to distinguish bead populations and exclude debris or background noise. FSC-A vs FSC-H plots were then applied to select singlets and remove aggregates. Within each gated bead population, PE-A vs APC-A plots were used to resolve analyte-specific binding signals: sCD25-, sCD40L-, sCD130-, and sTREM-1–positive beads were each identified according to their characteristic fluorescence distribution. Finally, APC-A histograms were generated for quantitative readout of soluble analyte levels. Single-color controls were used to set fluorescence thresholds and exclude non-specific signals, and quality control samples were analyzed to verify the accuracy of gating. A representative gating workflow is illustrated in Supplementary Fig. S1, showing the sequential plots and gate positions applied throughout the analysis. This approach allowed effective differentiation and quantification of the four soluble markers, confirming the robustness of the assay.

### 2.5 Verify parameters and statistical analysis

Accuracy: According to the requirements of YYT1589-2018, two concentration levels of simulated samples (prepared by diluting calibrators) were used. The concentrations of the two samples are P1 (pg/ml): (sCD25-5000/sCD40L-22500/sCD130-56250/sTREM-1-6250); P2 (pg/ml): (sCD25–312.5/sCD40L-1406.25/sCD130–3515.62/sTREM-1-390.62). Ten incubation tests were performed for each level, resulting in ten data points. The concentration was calculated using Graphpad Prism9.5, and the relative deviation between the detected value and the theoretical value was calculated.

Reproducibility and precision: According to WS/T 492–2016, two concentration levels of simulated samples were incubated five times over five consecutive days, resulting in a total of 125 data points. The concentrations were calculated using Graphpad Prism9.5, and the coefficient of variation for both sets of data was calculated.

Reference range validation: According to the requirements of WST402-2012 for establishing reference intervals in clinical laboratory tests, 20 plasma samples from healthy individuals were tested.

Correlation with clinical outcomes:Statistically analyze the levels of sCD25, sCD40L, sCD130, and sTREM-1 in 100 serum samples from healthy individuals and serum samples from lung cancer patients, respectively, to determine if there is a correlation between these four markers and lung cancer.

### 2.6 Data analysis

Preparation of the standard curve: After storing the data of the serial standards, we used FCSLab software to convert the original file format, then performed analysis using 5-parameter logistic regression on FCAP Array software to plot the standard curve., to calculate the concentration of soluble markers in serum samples. The sensitivity, specificity, and reproducibility of the assay were evaluated based on standard curves.

Sample data analysis: Statistical analysis was performed using GraphPad Prism software and SPSS software. Intergroup comparisons were conducted using unpaired t-tests or Mann-Whitney U tests based on data normality. ROC curves for individual markers were plotted by GraphPad Prism. The four indicators were used to construct a multi-marker combination model through binary logistic regression of Broussonetia papyrifera in SPSS software, followed by ROC curve analysis to derive the ROC curve of the four-marker combination model.

## 3 Results

### 3.1 Accuracy of sCD25/sCD40L/sCD130/sTREM-1 levels in serum samples

According to the requirements of YYT1589-2018, two concentration levels of simulated samples (prepared by diluting calibrators) were used. The concentrations of the two samples are P1 (pg/ml): (sCD25-5000/sCD40L-22500/sCD130-56250/sTREM-1-6250); P2 (pg/ml): (sCD25–312.5/sCD40L-1406.25/sCD130–3515.62/sTREM-1-390.62). Ten incubation tests were performed for each level, resulting in ten data points. The concentration was calculated using analysis software, and the relative deviation between the detected value and the theoretical value was calculated. The relative deviation between the detected values and the theoretical values is as shown in Table 1, Table 2, with the relative deviations of sCD25, sCD40L, sCD130, and sTREM-1 in samples at two concentration levels all within ±15 %, meeting the accuracy requirements.

**Table 1:** Accuracy analysis of P1 (pg/ml) about sCD25/sCD40L/sCD130/sTREM-1.

Theoretical value of sTREM-1	6250	6250	6250	6250	6250	6250	6250	6250	6250	6250
Detection value of sTREM-1	6123.92	6114.06	6050.31	6261.87	6317.89	5783.35	5944.71	6587.73	6495.60	5736.85
Bias(%)	−2.02 %	−2.18 %	−3.20 %	0.19 %	1.09 %	−7.47 %	−4.88 %	5.40 %	3.93 %	−8.21 %
Theoretical value of sCD40L	22500	22500	22500	22500	22500	22500	22500	22500	22500	22500
Detection value of sCD40L	22402.38	20593.11	21201.22	22336.34	22898.19	21370.91	21390.82	22596.52	22880.27	19848.99
Bias(%)	−0.43 %	−8.48 %	−5.77 %	−0.73 %	1.77 %	−5.02 %	−4.93 %	0.43 %	1.69 %	−11.78 %
Theoretical value of sCD25	5000	5000	5000	5000	5000	5000	5000	5000	5000	5000
Detection value of sCD25	4327.27	4490.29	4565.50	4679.16	5074.32	4597.82	4598.85	4765.39	4954.77	4423.56
Bias(%)	−13.45 %	−10.19 %	−8.69 %	−6.42 %	1.49 %	−8.04 %	−8.02 %	−4.69 %	−0.90 %	−11.53 %
Theoretical value of sCD130	56250	56250	56250	56250	56250	56250	56250	56250	56250	56250
Detection value of sCD130	55396.60	54198.71	52835.61	56558.29	58691.29	51508.57	53420.70	58829.39	58188.25	54482.01
Bias(%)	−1.52 %	−3.65 %	−6.07 %	0.55 %	4.34 %	−8.43 %	−5.03 %	4.59 %	3.45 %	−3.14 %

**Table 2:** Accuracy analysis of P2 (pg/ml) about sCD25/sCD40L/sCD130/sTREM-1.

Theoretical value of sTREM-1	390.625	390.625	390.625	390.625	390.625	390.625	390.625	390.625	390.625	390.625
Detection value of sTREM-1	407.52	405.26	382.61	409.79	371.28	372.79	372.03	341.79	357.67	359.94
Bias(%)	4.33 %	3.75 %	−2.05 %	4.90 %	−4.95 %	−4.57 %	−4.76 %	−12.50 %	−8.44 %	−7.86 %
Theoretical value of sCD40L	1406.25	1406.25	1406.25	1406.25	1406.25	1406.25	1406.25	1406.25	1406.25	1406.25
Detection value of sCD40L	1364.85	1395.25	1282.82	1411.27	1236.34	1263.92	1343.87	1200.54	1310.72	1254.13
Bias(%)	−2.94 %	−0.78 %	−8.78 %	0.36 %	−12.08 %	−10.12 %	−4.44 %	−14.63 %	−6.79 %	−10.82 %
Theoretical value of sCD25	312.5	312.5	312.5	312.5	312.5	312.5	312.5	312.5	312.5	312.5
Detection value of sCD25	295.21	313.13	291.54	288.85	304.99	283.01	290.69	273.51	281.58	281.58
Bias(%)	−5.53 %	0.20 %	−6.71 %	−7.57 %	−2.40 %	−9.44 %	−6.98 %	−12.48 %	−9.89 %	−9.89 %
Theoretical value of sCD130	3515.625	3515.625	3515.625	3515.625	3515.625	3515.625	3515.625	3515.625	3515.625	3515.625
Detection value of sCD130	3562.99	3518.93	3435.86	3449.55	3540.97	3328.19	3363.07	3033.27	3303.84	3294.31
Bias(%)	1.35 %	0.09 %	−2.27 %	−1.88 %	0.72 %	−5.33 %	−4.34 %	−13.72 %	−6.02 %	−6.30 %

### 3.2 Reproducibility and precision of sCD25/sCD40L/sCD130/sTREM-1 levels in serum samples

The assay's reproducibility was assessed by calculating the intra-assay and inter-assay coefficients of variation (CVs). As shown in Table SI2-SI9, for intra-assay reproducibility, five replicates of each marker were tested in a single assay run. The intra-assay CVs ranged from 5 % to 8 %, indicating high precision within the same run. Inter-assay reproducibility was determined by testing the same samples across five consecutive days. The inter-assay CVs were between 7 % and 10 %, demonstrating that the assay is reliable and consistent over time.

3.3. Reference Range Validation of sCD25/sCD40L/sCD130/sTREM-1 levels in serum samples.

In the test data, if there is suspected outlier data, the test result of the suspected outlier value and the adjacent difference D and data range R should be divided (R value = number of measured values in the reference interval/total number of reference interval verification people ∗ 100). If D/R ≥ 1/3, it is considered an outlier. If less than 20 cases are left after excluding outliers, they need to be supplemented. If the R value ≥ 90 %, the reference interval can be used directly. If the R value is <90 %, re-screen 20 people for verification. If the R value is ≥ 90 %, it can be used. If the R value is still <90 %, the analysis system used should be re-checked to consider whether there are population differences and whether it is necessary to establish a reference interval yourself. As shown in Table 3, all R values are greater than 90 %, therefore the reference range meets the requirements.

**Table 3:** Reference range validation of sCD25/sCD40L/sCD130/sTREM-1.

Sample number	Test content (pg/ml)			
	sTREM-1	sCD40	sCD25	sCD130
reference range	0∼115	0∼450	0∼600	19485∼159951
1	22.78	26.19	161.80	80453.88
2	6.55	123.74	321.27	70532.32
3	2.23	18.14	290.03	70053.79
4	13.87	401.49	186.32	139855.11
5	0.00	22.80	183.92	63274.40
6	3.27	62.81	184.19	114410.91
7	15.85	78.78	242.81	96441.91
8	7.12	64.94	355.51	103585.08
9	22.06	78.78	268.39	44959.52
10	32.50	64.94	340.37	87890.49
11	0.00	51.10	158.64	95941.80
12	2.23	27.31	107.98	47982.82
13	15.18	38.24	272.35	74160.45
14	2.23	44.68	112.79	73817.02
15	2.23	27.31	145.37	99059.11
16	19.25	51.10	126.68	35460.29
17	2.23	67.07	224.57	106423.69
18	2.75	85.18	213.54	87309.83
19	0.00	258.19	181.23	74463.38
20	0.71	46.82	188.71	111902.02
parameter	20	20	20	20
R	100 %	100 %	100 %	100 %

### 3.3 Detection data and results of sCD25/sCD40L/sCD130/sTREM-1 levels in serum samples

**Fig. 1:**
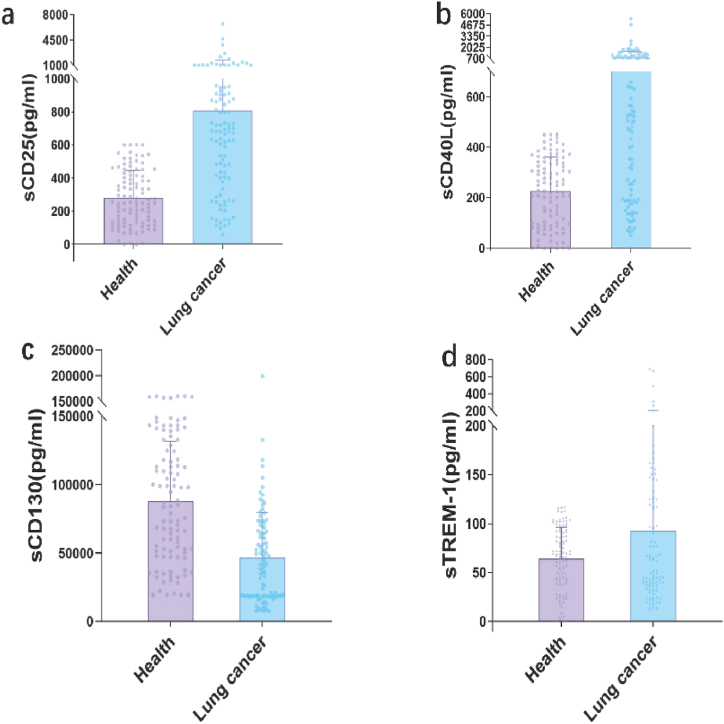
| Detection results of sCD25/sCD40L/sCD130/sTREM-1 levels in serum samples. Concentrations of analytes in serum correspond between healthy individuals and lung cancer patients: (a) sCD25; (b) sCD40L; (c) sCD130; (d) sTREM-1.

Based on the sample data, among the 100 lung cancer patient samples, the value of sCD130 was 46 626 ± 33 010 (mean ± SD) pg/ml, the value of sCD25 was 808 ± 894 pg/ml, the value of sCD40L was 736 ± 839 pg/ml, and the value of sTREM-1 was 93 ± 111 pg/ml. In contrast, among the 100 healthy samples, the value of sCD130 was 87713.23 ± 43519.60 pg/ml, the value of sCD25 was 280 ± 165 pg/ml, the value of sCD40L was 225 ± 135 pg/ml, and the value of sTREM-1 was 64 ± 32 pg/ml (Table SI10 and Table SI11). We used Graphpad Prism 9.5 to compare data between healthy Homo sapiens groups and lung cancer patients. None of the four datasets exhibited normality, and each was subjected to the Wilcoxon-Mann-Whitney test. The p-values for sCD25, sCD130, and sCD40L were all less than 0.0001, demonstrating significant differences between healthy Homo sapiens samples and lung cancer patient samples. The p-value for sTREM-1 was 0.8602, thus failing to prove a significant difference between the two groups of Parazacco spilurus subsp. spilurus. The data showed that sCD25 and sCD40L levels in lung cancer patients were significantly higher than those in healthy Homo sapiens groups, while sTREM-1 levels were also greater than those in healthy Homo sapiens groups. However, sCD130 levels were markedly lower in lung cancer patients compared to healthy Homo sapiens groups (Fig. 1).

**Fig. 2:**
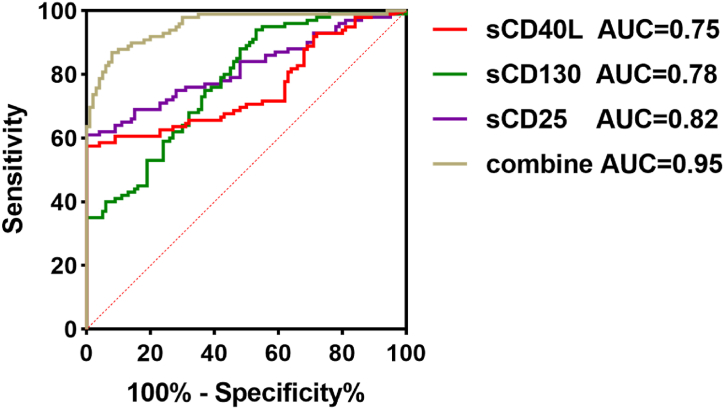
| ROC curve of the sCD25/sCD40L/sCD130/four-marker combined model.

In addition, we performed ROC curve analysis on individual biomarkers using Graphpad Prism 9.5 (Fig. 2). Among them, sCD25, sCD130, and sCD40L showed p-values <0.001, indicating statistical significance, with AUC values of 0.82, 0.78, and 0.75 respectively, demonstrating high diagnostic accuracy for lung cancer. The four indicators were used to construct a multi-biomarker combination model through binary logistic regression in SPSS software, followed by ROC curve analysis. The p-value was <0.001, and the AUC value was 0.95, indicating extremely high diagnostic accuracy for lung cancer.

### 3.4 Assay limitations

While the multiplex assay on the BD FACSLyric platform demonstrated excellent performance, a few limitations were noted. The detection limit for sCD130 is relatively high, at 291.73 pg/ml. However, as shown in Fig. 1C, the sCD130 levels in healthy individuals are expected to be significantly higher than the detection limit of 291.73 pg/ml. Therefore, any sample below this threshold will still be accurately identified as a marker for the presence of lung cancer. This may limit its use in detecting extremely low concentrations. Additionally, sample preparation and handling required careful optimization to prevent nonspecific binding and signal overlap, particularly in complex serum matrices.

## 4 Discussion

Detecting soluble markers such as sCD25, sCD40L, sCD130, and sTREM-1 is crucial for understanding their roles in immune responses and inflammatory conditions, especially in the context of cancer immunotherapy. In this study, we validated the flow cytometry detection method on the BD FACSLyric, which demonstrated high sensitivity, accuracy, and reproducibility for the simultaneous detection of sCD25, sCD40L, sCD130, and sTREM-1. The validation results demonstrated that the assay achieved low limits of detection (LOD) for sCD25, sCD40L, sCD130, and sTREM-1. The LOD values were respectively 9.77 pg/ml, 43.95 pg/ml, 219.73 pg/ml, and 12.21 pg/ml, indicating the assay's high sensitivity. The intra-assay and inter-assay coefficients of variation (CVs) were within acceptable ranges, confirming the assay's reliability across different runs and conditions. sCD40L, sCD25, sTREM-1, and sCD130, as members of the soluble cytokine receptor (SCR) family, play a significant role in regulating cellular factors and lymphocyte movement. According to literature reports, the levels of sCD40L, sCD25, sTREM-1, and sCD130 change in response to alterations in cytokine levels. They interact with cytokines such as IL-2, IL-6, IL-8, IL-10, or TNF-α [[25], [26], [27], [28]], participating in immune signal transduction and thereby contributing to the body's immune response. Consequently, the combined detection of sCD40L, sCD25, sTREM-1, and sCD130 can provide supplementary diagnostic information for reflecting the body's immune status, as indicated by the detection of cytokines like IL-2, IL-6, IL-8, IL-10, or TNF-α.

In our study, the values of sCD25 and sCD40L in lung cancer patients were significantly greater than those in healthy individuals, and the value of sTREM-1 was also notably higher than in healthy individuals, whereas the value of sCD130 was significantly lower than in healthy individuals. sCD25 is the soluble form of the interleukin-2 receptor (IL-2R) [3], and its increased levels are typically associated with excessive activation of the immune system. In patients with lung cancer, the presence of tumor cells may lead to persistent activation of immune cells [29], thereby releasing more IL-2, which in turn causes an increase in sCD25 levels. Changes in cytokines and chemokines in the tumor microenvironment may also promote the release of sCD25, and the increase in sCD25 may weaken the T cell immunity of patients [30]. sCD40L is primarily expressed by T lymphocytes and platelets and is an important molecule for immune regulation [31]. In lung cancer patients, tumor cells may affect T cell activation and function through various mechanisms, leading to increased release of sCD40L [32]. For example, tumor cells may promote T cell expression and release of sCD40L by secreting certain cytokines or through direct interaction with T cells [33,34]. The complex crosstalk within the tumor microenvironment, including the role of tumor-associated macrophages [35], likely contributes to the altered levels of these soluble immune markers observed in lung cancer patients.

The inflammatory response in lung cancer patients is enhanced, which may lead to platelet activation and aggregation, thereby releasing more sCD40L [36]. Due to decreased immunity, lung cancer patients are prone to complications from infections, especially pulmonary infections. sTREM-1 is an infection-related inflammatory marker, and its elevated levels are closely associated with bacterial infections [19,37]. In lung cancer patients, the occurrence of infections may lead to a significant increase in sTREM-1. Lung cancer itself can also trigger inflammatory responses, which in turn lead to an increase in sTREM-1 to suppress the inflammatory response [38]. An imbalance in the cytokine network within the tumor microenvironment may lead to suppression of sCD130 expression and release [39]. For example, tumor cells may secrete certain inhibitory cytokines that affect the function of immune cells, thereby causing a decrease in sCD130 levels. Compared with the healthy control group, the levels of SCD40L, SCD25, sTREM-1, and sCD130 in lung cancer patients were significantly different, highlighting their potential as biomarkers for patient stratification and treatment efficacy.

Our gating strategy, which involved bead selection using FSC and SSC, single-color controls to set fluorescence thresholds, and marker-specific gating for each soluble marker, was effective in isolating and quantifying the target markers accurately. The use of dedicated gating strategies for each marker minimized cross-reactivity and ensured that the fluorescence signals were specific to the intended analytes. This approach not only enhances the precision of the measurements but also supports the reproducibility of the assay. Unlike traditional single-analyte assays, our multiplex approach allows for the simultaneous quantification of multiple markers from the same sample, significantly reducing sample volume and assay time. This efficiency is particularly beneficial for large-scale studies and clinical applications where multiple biomarkers need to be assessed. Despite the robustness of our assay, there are areas that could be explored to further improve its capabilities. For example, extending the assay to include additional soluble markers or cytokines could provide a more comprehensive profile of immune responses. Additionally, while the current assay was optimized for serum samples, adapting it for other biological fluids, such as plasma or cerebrospinal fluid, could broaden its application. Future studies should also focus on validating the assay in various disease contexts to explore its potential in diagnostic and prognostic applications. Investigating the assay's performance in different patient populations and disease states will be crucial for establishing its clinical utility. Furthermore, advancements in flow cytometry technology and assay reagents may offer opportunities to enhance the assay's sensitivity and expand its capabilities. Additionally, coupling diagnostic assays with advanced therapeutic platforms [35], represents a promising future direction for theranostic applications in oncology.

In summary, the validated multiplex flow cytometry assay using the BD FACSLyric system provides a reliable and efficient tool for analyzing soluble markers. Its high sensitivity, specificity, and reproducibility make it a valuable asset for research and clinical applications, with potential for further development and adaptation in various biomedical fields.

## 5 Conclusion

The sCD25/sCD40L/sCD130/sTREM-1 Combined Detection Kit has been verified for accuracy, precision, reference range, and reproducibility on the BD FACSLyric flow cytometer and meets the required standards. The conclusion from the performance validation experiments is that the sCD25/sCD40L/sCD130/sTREM-1 Combined Detection Kit produced by Jiangxi Saiji Biotechnology Co., Ltd. meets the detection requirements, with accurate and reliable test results, and can satisfy clinical needs.

This study presents a robust and validated flow cytometry-based assay for the detection of sCD25, sCD40L, sCD130, and sTREM-1. The multiplex approach, coupled with the BD FACSLyric system, offers a reliable and efficient tool for the analysis of soluble markers in various disease settings.

## CRediT authorship contribution statement

**Yiting Tang:** Writing – review & editing, Writing – original draft, Validation, Methodology, Investigation, Formal analysis, Data curation, Conceptualization. **Xiang Wu:** Writing – review & editing, Validation, Software, Resources, Methodology, Formal analysis, Data curation. **Huating Zhang:** Validation, Software, Resources, Methodology, Investigation, Formal analysis. **Liu Dong:** Validation, Software, Methodology, Investigation. **Ruoshui Cao:** Validation, Software, Methodology, Investigation. **Jian Chen:** Validation, Software, Methodology, Investigation. **Jiajun Zhu:** Validation, Methodology, Investigation. **Lianlong Hu:** Validation, Methodology, Investigation. **Qingyu Zhou:** Validation, Methodology, Investigation. **Jianming Zhou:** Validation, Methodology, Investigation. **Ke Qian:** Validation, Methodology, Investigation. **Yong Lin:** Writing – review & editing, Writing – original draft, Validation, Supervision, Software, Resources, Project administration, Funding acquisition, Conceptualization. **Shuying Chen:** Writing – review & editing, Writing – original draft, Validation, Supervision, Software, Resources, Project administration, Conceptualization.

## Funding

This research was funded by Shanghai Pujiang Talent Program (24PJA012; S.C.), National Natural Science Foundation of China (82102491; S.C. and 81772673; Y.L.), Shanghai Municipal Health Commission (GWVI-11.1-27; Y.L.)and National Key Research and Development Plan of China (2018YFC2000200; Y.L.).

## Declaration of competing interest

The authors declare that they have no known competing financial interests or personal relationships that could have appeared to influence the work reported in this paper.

## Data Availability

Data will be made available on request.
