# Supplementary material for: Flow cytometry-based validation of soluble biomarker detection
Source: Pract Lab Med. 2025 Nov 7;47:e00510. doi: 10.1016/j.plabm.2025.e00510 (PMC12648499; doi:10.1016/j.plabm.2025.e00510)
Supplement: Multimedia component 1 [file mmc1.docx]

**Supplementary Materials**

**Flow cytometry-based validation of soluble biomarker detection**

**Table SI1** Configuration of BD FACSLyric Flow Cytometer

| **Laser (nm)** | **Filter** | **Representative Fluorochromes** |
| --- | --- | --- |
| Blue (488) | 527/32 (507LP) | FITC, AF488, BB515, GFP |
|  | 586/42 (560LP) | PE, PI |
|  | 700/54 (665LP) | PerCP, PerCP-Cy5.5, 7-AAD, BV700 |
|  | 783/56 (752LP) | PE-Cy7 |
| Red (640) | 660/10 (660/10) | APC, AF647 |
|  | 720/30 (705LP) | APC-R700, AF700 |
|  | 783/56 (752LP) | APC-Cy7, APC-H7 |
| Violet (405) | 448/45 | Pacific Blue, DAPI, V450, BV421 |
|  | 528/45 (500LP) | AmCyan, V500, V500-C, BV510 |
|  | 606/36 (606/36) | BV605 |
|  | 660/36 (706/36) | BV711 |
|  | 715/50 (715/50) | BV785 |
|  | 755LP | BV786 |

**Table SI2** Inter-batch CV and Intra-batch CV of sTREM-1 for P1

| Day X | Result1 | Result2 | Result3 | Result4 | Result5 | Intra-batch CV |
| --- | --- | --- | --- | --- | --- | --- |
| 1 | 6123.92 | 6114.06 | 6050.31 | 6261.87 | 6317.89 | 1.81% |
| 2 | 5588.76 | 5565.06 | 5969.03 | 6051.07 | 5639.18 | 3.98% |
| 3 | 5431.95 | 5404.75 | 5636.98 | 5660.83 | 6168.14 | 5.42% |
| 4 | 5482.21 | 5257.66 | 5372.75 | 5357.18 | 6233.73 | 7.14% |
| 5 | 5588.47 | 5541.34 | 6532.75 | 5761.75 | 5587.87 | 7.18% |
| Inter-batch mean（‾X） | 5787.98 | | | | | |
| Inter-batch standard deviation（SD） | 358.90 | | | | | |
| Inter-batch CV | 6.20% | | | | | |

**Table SI3** Inter-batch CV and Intra-batch CV of sTREM-1 for P2

| Day X | Result1 | Result2 | Result3 | Result4 | Result5 | Intra-batch CV |
| --- | --- | --- | --- | --- | --- | --- |
| 1 | 389.05 | 391.33 | 375.35 | 408.41 | 357.03 | 5.00% |
| 2 | 411.46 | 436.77 | 399.81 | 410.49 | 373.69 | 5.61% |
| 3 | 394.23 | 401.52 | 403.60 | 415.04 | 416.08 | 2.30% |
| 4 | 407.52 | 405.26 | 382.61 | 409.79 | 371.28 | 4.38% |
| 5 | 372.79 | 372.03 | 341.79 | 357.67 | 359.94 | 3.51% |
| Inter-batch mean | 390.58 | | | | | |
| Inter-batch standard deviation | 23.22 | | | | | |
| Inter-batch CV | 5.94% | | | | | |

**Table SI4** Inter-batch CV and Intra-batch CV of sCD25 for P1

| Day X | Result1 | Result2 | Result3 | Result4 | Result5 | Intra-batch CV |
| --- | --- | --- | --- | --- | --- | --- |
| 1 | 4327.27 | 4490.29 | 4565.50 | 4679.16 | 5074.32 | 6.07% |
| 2 | 4493.11 | 4457.73 | 4461.14 | 5190.55 | 4336.50 | 7.46% |
| 3 | 4616.59 | 4335.12 | 4684.40 | 4760.53 | 4637.00 | 3.51% |
| 4 | 4779.57 | 4659.95 | 4461.00 | 4417.58 | 5003.29 | 5.15% |
| 5 | 4511.20 | 4787.18 | 4961.09 | 4794.38 | 4797.98 | 3.40% |
| Inter-batch mean | 4651.30 | | | | | |
| Inter-batch standard deviation | 235.25 | | | | | |
| Inter-batch CV | 5.06% | | | | | |

**Table SI5** Inter-batch CV and Intra-batch CV of sCD25 for P2

| Day X | Result1 | Result2 | Result3 | Result4 | Result5 | Intra-batch CV |
| --- | --- | --- | --- | --- | --- | --- |
| 1 | 299.09 | 316.91 | 305.93 | 310.89 | 287.48 | 3.73% |
| 2 | 336.56 | 313.42 | 317.88 | 321.09 | 326.27 | 2.75% |
| 3 | 321.46 | 312.07 | 331.30 | 334.43 | 307.34 | 3.66% |
| 4 | 295.21 | 313.13 | 291.54 | 288.85 | 304.99 | 3.38% |
| 5 | 283.01 | 290.69 | 273.51 | 281.58 | 281.58 | 2.16% |
| Inter-batch mean | 305.85 | | | | | |
| Inter-batch standard deviation | 17.92 | | | | | |
| Inter-batch CV | 5.86% | | | | | |

**Table SI6** Inter-batch CV and Intra-batch CV of sCD40L for P1

| Day X | Result1 | Result2 | Result3 | Result4 | Result5 | Intra-batch CV |
| --- | --- | --- | --- | --- | --- | --- |
| 1 | 22402.38 | 20593.11 | 21201.22 | 22336.34 | 22898.19 | 4.36% |
| 2 | 20309.09 | 20798.24 | 22645.22 | 21284.16 | 19942.80 | 5.01% |
| 3 | 22168.00 | 22057.69 | 23372.10 | 22305.62 | 23815.37 | 3.50% |
| 4 | 21716.17 | 22067.63 | 21365.57 | 20358.73 | 23141.76 | 4.67% |
| 5 | 21368.70 | 23454.52 | 24143.47 | 22100.51 | 21504.54 | 5.46% |
| Inter-batch mean | 21974.05 | | | | | |
| Inter-batch standard deviation | 1123.59 | | | | | |
| Inter-batch CV | 5.11% | | | | | |

**Table SI7** Inter-batch CV and Intra-batch CV of sCD40L for P2

| Day X | Result1 | Result2 | Result3 | Result4 | Result5 | Intra-batch CV |
| --- | --- | --- | --- | --- | --- | --- |
| 1 | 1270.85 | 1347.77 | 1311.23 | 1391.81 | 1233.51 | 4.75% |
| 2 | 1519.16 | 1435.28 | 1347.80 | 1491.07 | 1332.55 | 3.98% |
| 3 | 1312.67 | 1322.83 | 1335.52 | 1311.82 | 1340.58 | 0.99% |
| 4 | 1364.85 | 1395.25 | 1282.82 | 1411.27 | 1236.34 | 5.64% |
| 5 | 1263.92 | 1343.87 | 1200.54 | 1310.72 | 1254.13 | 4.32% |
| Inter-batch mean | 1346.73 | | | | | |
| Inter-batch standard deviation | 68.28 | | | | | |
| Inter-batch CV | 5.07% | | | | | |

**Table SI8** Inter-batch CV and Intra-batch CV of sCD130 for P1

| Day X | Result1 | Result2 | Result3 | Result4 | Result5 | Intra-batch CV |
| --- | --- | --- | --- | --- | --- | --- |
| 1 | 55396.60 | 54198.71 | 52835.61 | 56558.29 | 58691.29 | 4.04% |
| 2 | 50430.14 | 52545.14 | 55345.19 | 55870.73 | 53757.51 | 4.11% |
| 3 | 51743.67 | 49498.18 | 53155.14 | 51145.76 | 53657.47 | 3.20% |
| 4 | 53237.89 | 53717.53 | 56026.23 | 51360.78 | 57339.90 | 4.35% |
| 5 | 51424.96 | 52211.74 | 59524.84 | 55123.45 | 55259.05 | 5.83% |
| Inter-batch mean | 54002.23 | | | | | |
| Inter-batch standard deviation | 2521.15 | | | | | |
| Inter-batch CV | 4.67% | | | | | |

**Table SI9** Inter-batch CV and Intra-batch CV of sCD130 for P2

| Day X | Result1 | Result2 | Result3 | Result4 | Result5 | Intra-batch CV |
| --- | --- | --- | --- | --- | --- | --- |
| 1 | 3260.50 | 3483.31 | 3316.97 | 3223.82 | 3060.79 | 4.68% |
| 2 | 3757.17 | 4027.97 | 3530.83 | 3550.14 | 3426.93 | 6.52% |
| 3 | 3606.77 | 3401.88 | 3753.57 | 3732.84 | 3484.30 | 4.26% |
| 4 | 3562.99 | 3518.93 | 3435.86 | 3449.55 | 3540.97 | 1.61% |
| 5 | 3328.19 | 3363.07 | 3033.27 | 3303.84 | 3294.31 | 4.04% |
| Inter-batch mean | 3457.95 | | | | | |
| Inter-batch standard deviation | 220.71 | | | | | |
| Inter-batch CV | 6.38% | | | | | |

**Table SI10** Detection data and results of sCD25/sCD40L/sCD130/sTREM-1 levels in serum samples for healthy person

|  | sCD25 | sCD40L | sCD130 | sTREM-1 |
| --- | --- | --- | --- | --- |
| 1 | 219.7 | 384.75 | 83569.18 | 81.01 |
| 2 | 364.61 | 151.21 | 19485 | 0 |
| 3 | 103.23 | 373.46 | 68074.77 | 76.34 |
| 4 | 188.22 | 421.7 | 47101.98 | 20.72 |
| 5 | 128.75 | 90.24 | 28722.97 | 40.52 |
| 6 | 410.09 | 54.39 | 51383.97 | 103.73 |
| 7 | 295.67 | 79.84 | 135360.71 | 17.76 |
| 8 | 242.69 | 293.9 | 47198.12 | 95.08 |
| 9 | 600 | 199.74 | 66277.94 | 27.37 |
| 10 | 253.58 | 201.56 | 143244.27 | 65.71 |
| 11 | 555.94 | 209.08 | 108377.59 | 48.74 |
| 12 | 273.43 | 269.64 | 94399.47 | 92.2 |
| 13 | 108.96 | 147.73 | 50503.18 | 102.35 |
| 14 | 382.27 | 331.1 | 148661.81 | 38.29 |
| 15 | 452.84 | 358.41 | 67918.69 | 75.12 |
| 16 | 450.61 | 306.37 | 98061.86 | 71.55 |
| 17 | 416.44 | 223.11 | 148885.52 | 96.39 |
| 18 | 466.68 | 358.84 | 139181.82 | 102.08 |
| 19 | 204.22 | 341.79 | 132819.01 | 68.84 |
| 20 | 343.09 | 82.54 | 109781.3 | 26.79 |
| 21 | 243.2 | 358.23 | 143185.29 | 87.13 |
| 22 | 452.86 | 368.46 | 132819.93 | 38.64 |
| 23 | 333.77 | 57.85 | 141819.29 | 53.06 |
| 24 | 391.05 | 253.36 | 53403.58 | 55.46 |
| 25 | 248.85 | 305.41 | 148521.52 | 23.31 |
| 26 | 310.1 | 291.89 | 103682.67 | 98.13 |
| 27 | 441.57 | 248 | 66437.73 | 105.37 |
| 28 | 351.6 | 134.74 | 75626.62 | 23.23 |
| 29 | 538.1 | 50.61 | 124914.47 | 112.8 |
| 30 | 146.23 | 74.07 | 19485 | 68.72 |
| 31 | 461.71 | 265.13 | 64631.17 | 42 |
| 32 | 88.14 | 389.92 | 157434.92 | 112.44 |
| 33 | 4.4 | 280.47 | 112721.67 | 25.46 |
| 34 | 402.39 | 360.38 | 46500.14 | 4.32 |
| 35 | 600.01 | 384.88 | 158815.36 | 91.14 |
| 36 | 209.08 | 87.32 | 107596.2 | 71.65 |
| 37 | 542.68 | 204.97 | 73662.18 | 98.44 |
| 38 | 290.17 | 89.22 | 22320.36 | 66.22 |
| 39 | 129.93 | 247.45 | 147236.48 | 1.73 |
| 40 | 551.69 | 411.49 | 112681.18 | 61.96 |
| 41 | 535.7 | 330.64 | 47414.25 | 66.88 |
| 42 | 334.26 | 28.21 | 140236.16 | 79.51 |
| 43 | 277.33 | 159.87 | 32332.24 | 69.43 |
| 44 | 287.58 | 0 | 156570.34 | 86.45 |
| 45 | 178.25 | 19.45 | 55337.29 | 92.15 |
| 46 | 22.13 | 21.57 | 128690.54 | 70.48 |
| 47 | 0 | 225.22 | 143366.14 | 115.67 |
| 48 | 231.65 | 200.43 | 104445.04 | 28.41 |
| 49 | 210.69 | 262.06 | 78907.43 | 101.56 |
| 50 | 248.49 | 373.75 | 145801.57 | 5.03 |
| 51 | 198.24 | 420.28 | 84096.04 | 93.56 |
| 52 | 125.6 | 207.34 | 36240.36 | 37.97 |
| 53 | 518.7 | 218.02 | 116596.36 | 69.01 |
| 54 | 132.66 | 77.39 | 33252.58 | 43.91 |
| 55 | 147.08 | 314.44 | 86927.9 | 8.29 |
| 56 | 67.84 | 62.92 | 48024.14 | 99.58 |
| 57 | 5.47 | 276.47 | 97858.72 | 44.16 |
| 58 | 342.84 | 253.33 | 53068.22 | 43.24 |
| 59 | 88.95 | 303.36 | 19484.9 | 49.61 |
| 60 | 87.07 | 220.48 | 61401.77 | 90.53 |
| 61 | 327.66 | 410.54 | 110002.19 | 22.76 |
| 62 | 471.53 | 228.5 | 19484.5 | 82.47 |
| 63 | 169.92 | 145.58 | 117975.99 | 85.69 |
| 64 | 502.79 | 429.94 | 159952.1 | 78.86 |
| 65 | 154.82 | 437.74 | 68727.81 | 71.53 |
| 66 | 600.35 | 450 | 35457.83 | 25.6 |
| 67 | 114.27 | 371.05 | 98746.36 | 60.2 |
| 68 | 484.31 | 98.33 | 53381.09 | 81.46 |
| 69 | 334.49 | 183.21 | 100148.81 | 116.58 |
| 70 | 600 | 0 | 33645.25 | 56.77 |
| 71 | 168.65 | 322.85 | 51503.23 | 81.57 |
| 72 | 195.12 | 0 | 58104.76 | 41.69 |
| 73 | 494.68 | 304.64 | 158886.13 | 103.28 |
| 74 | 430.5 | 104.14 | 60260.6 | 86.71 |
| 75 | 267.95 | 126.19 | 85080.76 | 99.22 |
| 76 | 490.16 | 345.78 | 30145.07 | 44.46 |
| 77 | 141.54 | 342.09 | 118349.24 | 115 |
| 78 | 365.27 | 58.16 | 32262.72 | 101.64 |
| 79 | 113.54 | 294.80 | 98861.94 | 14.79 |
| 80 | 113.87 | 30.25 | 98874.58 | 56.94 |
| 81 | 255.24 | 362.67 | 34922.32 | 103.70 |
| 82 | 0.00 | 74.70 | 113121.40 | 46.41 |
| 83 | 108.08 | 257.89 | 159951.05 | 60.54 |
| 84 | 90.45 | 207.67 | 54681.89 | 97.29 |
| 85 | 152.29 | 1.81 | 20652.60 | 18.98 |
| 86 | 518.67 | 115.08 | 124314.93 | 63.76 |
| 87 | 374.20 | 451.67 | 73238.68 | 71.48 |
| 88 | 383.87 | 100.97 | 36271.29 | 55.73 |
| 89 | 414.08 | 337.29 | 28126.75 | 49.65 |
| 90 | 76.15 | 1.47 | 37467.03 | 24.47 |
| 91 | 221.40 | 390.28 | 73232.26 | 0.00 |
| 92 | 68.12 | 8.81 | 88507.07 | 105.87 |
| 93 | 207.12 | 324.73 | 35877.62 | 65.34 |
| 94 | 246.67 | 167.36 | 113824.03 | 0.00 |
| 95 | 132.38 | 96.85 | 143275.28 | 53.43 |
| 96 | 69.02 | 450.00 | 70421.53 | 61.72 |
| 97 | 80.66 | 94.19 | 159951.00 | 111.66 |
| 98 | 453.44 | 0.00 | 59937.88 | 37.21 |
| 99 | 105.44 | 395.38 | 139960.71 | 68.93 |
| 100 | 254.08 | 296.93 | 141180.03 | 102.23 |
| $\overline{x}$ | 279.9186 | 225.3802 | 87713.2322 | 64.1285 |
| SD | 164.7560732 | 134.7509625 | 43519.60052 | 31.98709332 |

**Table SI11** Detection data and results of sCD25/sCD40L/sCD130/sTREM-1 levels in serum samples for patients with lung cancer

|  | sCD25 | sCD40L | sCD130 | sTREM-1 |
| --- | --- | --- | --- | --- |
| 1 | 845.52 | 1168.87 | 14334.75 | 67.12 |
| 2 | 700.55 | 505.16 | 14335.75 | 42.93 |
| 3 | 691.54 | 230.32 | 14336.75 | 44.74 |
| 4 | 861.71 | 188.1 | 14337.75 | 45.15 |
| 5 | 685.92 | 0.00 | 14338.75 | 64.66 |
| 6 | 1015.43 | 403.74 | 14339.75 | 97.71 |
| 7 | 617.95 | 127.37 | 14340.75 | 77.30 |
| 8 | 606.95 | 161.62 | 14341.75 | 51.43 |
| 9 | 236.33 | 1651.57 | 14342.75 | 12.23 |
| 10 | 255.64 | 530.04 | 14343.75 | 31.73 |
| 11 | 285.3 | 638.24 | 14344.75 | 36.93 |
| 12 | 148.32 | 1873.08 | 14345.75 | 19.41 |
| 13 | 296.4 | 526.52 | 14346.75 | 27.67 |
| 14 | 128.66 | 521.82 | 14347.75 | 18.02 |
| 15 | 414.48 | 1891.47 | 14348.75 | 68.20 |
| 16 | 126 | 465.99 | 14349.75 | 124.71 |
| 17 | 260.34 | 1475.17 | 14350.75 | 23.25 |
| 18 | 482.01 | 794.35 | 14351.75 | 43.03 |
| 19 | 402.17 | 1401.38 | 14352.75 | 40.12 |
| 20 | 235.27 | 62.95 | 14353.75 | 32.05 |
| 21 | 147.27 | 67.15 | 14354.75 | 18.31 |
| 22 | 57.71 | 188.44 | 14355.75 | 19.24 |
| 23 | 211.25 | 176.36 | 14356.75 | 21.87 |
| 24 | 147.78 | 142.94 | 14357.75 | 19.07 |
| 25 | 262.35 | 433.04 | 14358.75 | 40.24 |
| 26 | 161.75 | 270.01 | 14359.75 | 24.52 |
| 27 | 114.34 | 140.31 | 14360.75 | 12.23 |
| 28 | 476.16 | 266.26 | 14361.75 | 48.14 |
| 29 | 334.69 | 109.54 | 14362.75 | 14.28 |
| 30 | 113.58 | 79.1 | 14363.75 | 14.64 |
| 31 | 399.21 | 192.8 | 14364.75 | 33.50 |
| 32 | 264.59 | 172.97 | 14365.75 | 35.69 |
| 33 | 203.14 | 136.32 | 14366.75 | 12.25 |
| 34 | 156.56 | 331.65 | 14367.75 | 23.59 |
| 35 | 98.24 | 188.44 | 14368.75 | 13.19 |
| 36 | 1072.37 | 993.34 | 57209.82 | 31.66 |
| 37 | 1134.93 | 635.99 | 23788.76 | 41.53 |
| 38 | 628.24 | 1657.70 | 42446.00 | 23.93 |
| 39 | 875.21 | 803.85 | 41504.27 | 35.62 |
| 40 | 720.09 | 561.64 | 65729.87 | 44.46 |
| 41 | 1034.31 | 504.03 | 79174.46 | 39.56 |
| 42 | 953.31 | 1022.55 | 113511.06 | 36.03 |
| 43 | 722.17 | 1132.84 | 36190.02 | 24.01 |
| 44 | 681.64 | 642.64 | 45837.53 | 21.51 |
| 45 | 666.53 | 4699.57 | 50282.46 | 39.26 |
| 46 | 636.45 | 860.12 | 49014.51 | 49.63 |
| 47 | 720.51 | 881.09 | 64657.42 | 52.09 |
| 48 | 1003.91 | 2358.22 | 84993.93 | 29.38 |
| 49 | 1016.1 | 721.12 | 74199.49 | 40.59 |
| 50 | 1272.78 | 480.55 | 64390.13 | 45.25 |
| 51 | 1046.59 | 460.70 | 73701.60 | 66.61 |
| 52 | 2625.1 | 564.97 | 117988.77 | 37.82 |
| 53 | 683.21 | 759.43 | 48169.42 | 61.88 |
| 54 | 810.86 | 499.20 | 68803.67 | 76.55 |
| 55 | 678.23 | 592.52 | 88183.28 | 39.77 |
| 56 | 797.85 | 630.11 | 81909.48 | 68.30 |
| 57 | 4660.77 | 978.60 | 92489.66 | 65.73 |
| 58 | 623.28 | 1021.26 | 31484.59 | 81.43 |
| 59 | 878.06 | 1456.97 | 66523.06 | 95.63 |
| 60 | 911.39 | 990.20 | 70285.69 | 63.24 |
| 61 | 6746.39 | 1788.59 | 132555.47 | 45.49 |
| 62 | 903.3 | 2807.28 | 51500.80 | 37.91 |
| 63 | 732.2 | 823.95 | 77165.09 | 90.03 |
| 64 | 1040.48 | 5388.81 | 69833.84 | 91.95 |
| 65 | 3839.58 | 473.75 | 27066.49 | 39.24 |
| 66 | 733.61 | 545.91 | 56244.75 | 42.82 |
| 67 | 947.42 | 1754.11 | 54236.29 | 80.97 |
| 68 | 702.93 | 807.51 | 72355.52 | 44.09 |
| 69 | 1015.43 | 523.14 | 54499.22 | 62.52 |
| 70 | 633.72 | 753.4 | 56009.69 | 96.75 |
| 71 | 1272.86 | 1605.78 | 50202.38 | 49.28 |
| 72 | 924.97 | 859.6 | 64529.76 | 77.12 |
| 73 | 435.16 | 103.26 | 86478.54 | 155.32 |
| 74 | 908.41 | 252.46 | 73552.63 | 165.91 |
| 75 | 672.71 | <43.95 | 94386.15 | 261.82 |
| 76 | 1292.67 | 194.78 | 73241.88 | 306.17 |
| 77 | 868.92 | 312.87 | 61606.70 | 117.33 |
| 78 | 376.9 | 135.84 | 57215.53 | 171.98 |
| 79 | 796.9 | 1092.45 | 61611.14 | 161.21 |
| 80 | 1906.14 | 1459.75 | 98011.00 | 492.11 |
| 81 | 534.15 | 71.41 | 32923.94 | 686.70 |
| 82 | 433.72 | 1282.02 | 80305.41 | 119.14 |
| 83 | 959.03 | 657.81 | 105121.86 | 144.99 |
| 84 | 407.04 | 539.86 | 52451.09 | 124.71 |
| 85 | 488.05 | 209.44 | 48037.98 | 121.97 |
| 86 | 511.55 | 352.95 | 52479.86 | 126.78 |
| 87 | 588.35 | 319.43 | 89218.89 | 199.83 |
| 88 | 797.72 | 113.63 | 20918.31 | 201.83 |
| 89 | 728.18 | 107.28 | 38894.38 | 146.75 |
| 90 | 625.81 | 49.48 | 40825.22 | 115.07 |
| 91 | 534.22 | 350.78 | 34830.91 | 132.59 |
| 92 | 0.00 | 254.17 | 60732.15 | 666.67 |
| 93 | 861.97 | 137.66 | 26381.22 | 161.56 |
| 94 | 256.58 | 1434.15 | 42552.70 | 157.13 |
| 95 | 483.28 | 149.74 | 35643.89 | 150.68 |
| 96 | 1450.94 | 81.55 | 10888.02 | 150.36 |
| 97 | 902.54 | 399.92 | 45221.93 | 179.61 |
| 98 | 947.07 | 875.7 | 49539.59 | 116.51 |
| 99 | 2181.21 | 184.14 | 38262.90 | 124.53 |
| 100 | 1012.08 | 181.36 | 20904.22 | 144.24 |
| $\overline{x}$ | 807.5319 | 735.939596 | 44332.1754 | 90.9229 |
| SD | 893.9229067 | 838.9107151 | 29718.61098 | 110.1349708 |

**
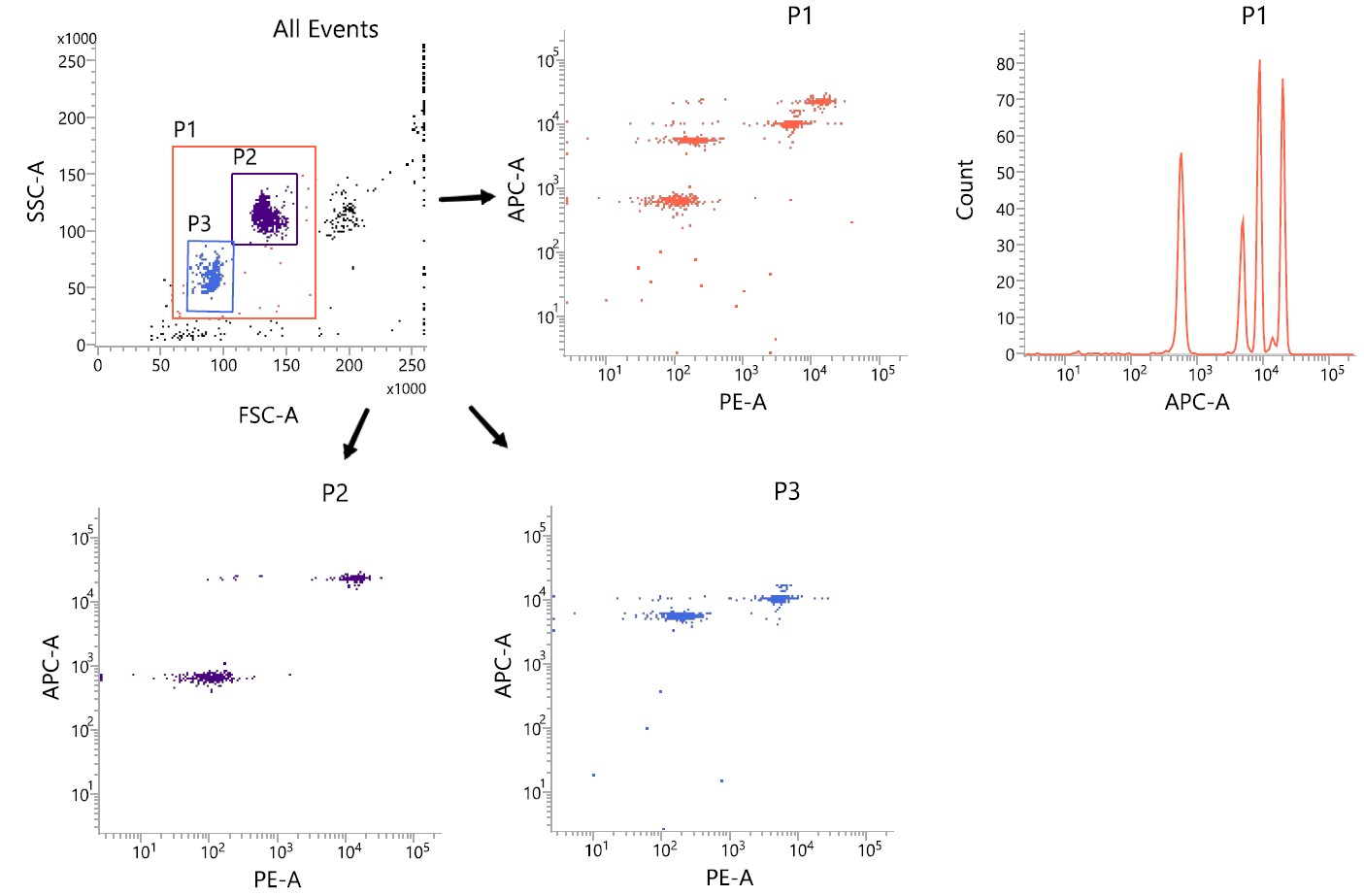
**

**Figure S1. Gating strategy for soluble analyte detection on BD FACSLyric.** Representative gating strategy applied to the multiplex bead-based assay for soluble analytes. Step 1: FSC-A vs SSC-A was used to identify bead populations (P1, P2, P3) and exclude debris. Step 2: Within each gated bead population, PE-A vs APC-A plots were used to resolve analyte-specific binding signals. Step 3: APC-A histograms were generated for quantitative readout of soluble analyte levels (e.g., in P1). This gating template was consistently applied across all samples to ensure reproducibility of the soluble analyte measurements
